# Analysis pipelines and packages for Infinium HumanMethylation450 BeadChip (450k) data

**DOI:** 10.1016/j.ymeth.2014.08.011

**Published:** 2015-01-15

**Authors:** Tiffany J. Morris, Stephan Beck

**Affiliations:** UCL Cancer Institute, University College London, London WC1E 6BT, UK

**Keywords:** HumanMethylation450, Epigenetics, Analysis pipelines, EWAS, DNA methylation, 450k BeadChip

## Abstract

The Illumina HumanMethylation450 BeadChip has become a popular platform for interrogating DNA methylation in epigenome-wide association studies (EWAS) and related projects as well as resource efforts such as the International Cancer Genome Consortium (ICGC) and the International Human Epigenome Consortium (IHEC). This has resulted in an exponential increase of 450k data in recent years and triggered the development of numerous integrated analysis pipelines and stand-alone packages. This review will introduce and discuss the currently most popular pipelines and packages and is particularly aimed at new 450k users.

## 1 Introduction

DNA methylation (DNAm) is an epigenetic modification that plays an important role in the regulation of gene expression and has become an important avenue of research in our quest to gain a better understanding of human development and disease. A number of lab techniques are available for interrogating DNAm [1] including the popular Infinium HumanMethylation450 BeadChip (450k array) [2], [3]. Although not as comprehensive as sequencing-based methods, it is more affordable and much simpler to analyse and interpret. These characteristics make the 450k array an ideal choice for epigenome-wide association studies (EWAS) involving hundreds or even thousands of cohort samples [4], [5], [6] and also for identifying methylation signatures as biomarkers of disease state and progression [7], [8].

The 450k array contains 485,512 probes covering 99% of RefSeq genes. The probes interrogate 19,755 unique CpG islands with additional coverage in shore regions and miRNA promoters as well as 3091 probes at non-CpG sites. For each probe sequence, a median of 14 beads is randomly distributed on the array. Each of these beads contains hundreds of thousands of oligonucleotides. This provides a unique set of internal technical replication on each array. The 450k array is an extension of an earlier BeadChip, the HumanMethylation27 array (27k array). The 27k array was designed using the Infinium I probe chemistry, which has two beads per probe, one in the red channel and one in the green channel. To create the more comprehensive array platform a second assay type, Infinium II, was used in addition to the Infinium I assay. The Infinium II assay only uses one bead per probe in the red or green channel representing methylated or unmethylated respectively. The Infinium II assay enabled more probes to fit on the array but due to particular probe characteristics (particularly CpG density within the probe) 30% of the probes retained the Infinium I assay creating the two-assay design of the 450k array. The two probe types display a slightly different dynamic range potentially leading to a type II bias during analysis [9].

**Fig. 1:**
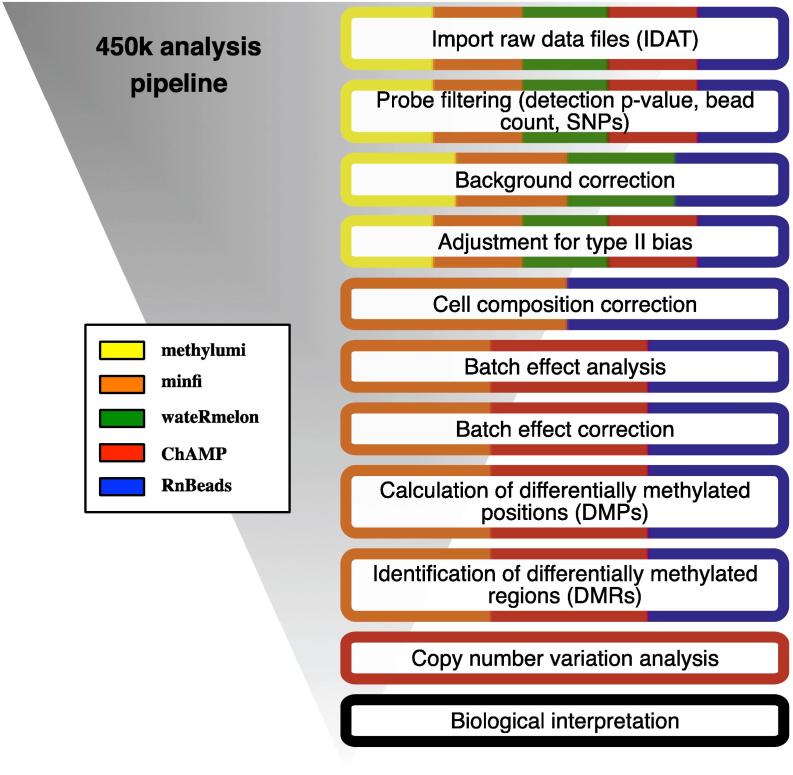
Popular 450k analysis pipelines with their respective module options.

This two-assay design has led to the development of a variety of specialised within-array normalisation algorithms [9], [10], [11], [12], [13], [14] to adjust for potential type II bias. This assay adjustment combined with other basic steps for preprocessing make up the general 450k array analysis workflow reviewed in detail by Dedeurwaerder et al. [15]. Briefly, raw data (IDAT files) is imported using the illuminaio [16] tool implemented in *minfi*. Then a number of quality control metrics are examined to determine the success of the bisulphite conversion and subsequent array hybridisation. Probe filtering is performed to remove probes that have failed to hybridise (detection *p*-value) and that are not represented by a minimum of 3 beads on the array. It may also be of interest to remove probes that overlap with single nucleotide polymorphisms (SNPs), that cross hybridise to multiple genomic locations or are on the sex chromosomes. After filtering it is important to perform within-array normalisation. Methods include background correction, dye-bias adjustment [17] and the aforementioned type II adjustment. Following this it is important to analyse data for potential batch effects. Batch effects [18] are a common source of variation in high-throughput experiments. They represent measurements related to conditions that are not the biological or scientific variables in the study (i.e. date of experiment, chip or instrument used, batch of reagents, technician running samples, etc.). Batch effects can usually be avoided with careful study design and the ability to correct for them is dependent on the degree of confounding in the particular dataset. Popular methods for batch effect correction are the supervised correction methods *ComBat* [19], surrogate variable analysis (SVA) [20] and independent surrogate variable analysis (ISVA) [21]. Also an unsupervised between-array normalisation method (i.e. functional normalisation [22]) may be effective in removing batch effects. Depending on the study, one may want to consider correction for cell heterogeneity [23]. After these preprocessing steps, the calculation of differentially methylated positions (DMPs) between groups of interest is the first step in the analysis process. This is most easily done between two groups, however, more complex analyses can be designed to look at time-course studies, twin designs [24], [25], [26], [27], multiple groups and paired samples. To focus analysis and narrow results calling differentially methylated regions (DMRs) is the next step [28]. Following this, additional steps in the 450k array analysis workflow include copy number alteration (CNA) analysis, integration with external data resources, data visualisation and data interpretation. There are a number of analysis packages that include many or all of these modules that make up the 450k workflow. The purpose of this review is to describe in detail five comprehensive, freely available 450k software packages (*methylumi*, *minfi*, *wateRmelon*, *ChAMP* and *RnBeads*). Fig. 1 shows the modules within these five analysis packages colour-coded to indicate which module is in which package. We will also highlight a number of stand-alone packages for additional downstream analysis (*Marmal-aid*, *RefFreeEWAS*, *EWasher*) that can be used in addition to the more comprehensive packages. The Illumina Genome Studio software only offers basic preprocessing and analysis options and requires the purchase of a license so will not be discussed in this review. Table 1 shows all free 450k software packages available to date.

**Table 1:** Freely available packages for Infinium 450k data analysis.

Package	Use	References
*ChAMP*	Comprehensive suite of functions; automated pipeline	[35]
*COHCAP*	CpG island analysis and gene expression data integration	[49]
*Comb-p*	DMR calling	[47]
*DMRcate*	DMR calling	[45]
Epigenetic clock	Predictor of sample age	[54]
*EWasher*	Reference-free cell composition correction	[53]
*FastDMA*	Quantile normalisation and DMP/DMR calling	[46]
IMA	Preprocessing including normalisation methods; Pipeline option	[60]
*Lumi*	Background correction, general normalisation	[61]
*Marmal-aid*	450k database for data integration	[48]
MethylAid	Interface for interactive sample QC	[39]
*Methylumi*	Comprehensive suite of functions	[29]
*Minfi*	Comprehensive suite of functions	[31]
*NIMBL*	Matlab code for QC and DMP calling	[62]
*RefFreeEWAS*	Reference-free cell composition correction	[37]
*RnBeads*	Comprehensive suite of functions	[36]
*shinyMethyl*	Interface for interactive sample QC	[38]
*wateRmelon*	Preprocessing including performance metrics and numerous normalisation methods	[12]

## 2 Comprehensive 450K analysis packages

Here we introduce five comprehensive packages developed for 450k array data analysis: *methylumi*, *minfi*, *wateRmelon*, *ChAMP* and *RnBeads*. The first four are available through Bioconductor while *RnBeads* is a stand-alone R-package. All five packages allow the user to import raw IDAT files or tabular methylation values. However, the availability of the IDAT files enables access to more functionality in each package (particularly quality control metrics).

### 2.1 *Methylumi*

The *methylumi* [29] Bioconductor package provides R classes for holding and manipulating 450k array data. These classes enable the access of MIAME (Minimum Information about a Microarray Experiment) information including sample details, feature information and multiple matrices of data. *Methylumi* enables the user to perform quality control interrogation, background correction and normalisation. In addition to 450k analysis *methylumi* has methods that work with GoldenGate and 27k array data.

*Methylumi* implements an effective normalisation option called noob (normal-exponential convolution using out-of-band probes) [30]. This method includes dye-bias equalisation to control for the different average intensities in the red and green channels and background correction to remove technical variation. Three models for background correction are included in *methylumi* and are used together with the intensities of either negative controls or out-of-band controls within each colour channel. Out-of-band probes are those that provide signals in the opposite channel from the probe design. There are 135,501 out-of-band probes on the 450k array. The noob normalisation uses normal-exponential convolution to subtract the background mean intensity (estimated from the out-of-band control probes) from the foreground intensity. Although noob was not specifically designed to correct for type II bias it is effective in doing so.

*Methylumi* has methods that convert its R classes to work with other 450k analysis packages allowing users to pick and choose modules from various packages that work best for a particular application.

### 2.2 *Minfi*

The *minfi* [31] Bioconductor package is a comprehensive package that like *methylumi* was developed early on and has its own set of R classes for holding and manipulating 450k array data. Although *minfi* does not provide a single function to run the entire pipeline it does offer all the modules required for a full workflow. It is frequently updated to offer methods for the newest analysis options available to 450k users. These include both DMR calling and block finding modules, a module for estimating cell-type composition of whole blood using a reference dataset [32] and a new between-array normalisation algorithm, termed functional normalisation [22].

*Minfi* has included the Genome Studio normalisation and preprocessing methods for users familiar with those methods and interested in using them. For quality control analysis a number of images are included and an HTML QC report can be created that includes visualisation of the array’s internal controls. It also has a useful function for predicting sample gender.

Two different functions in *minfi* estimate DMRs depending on the genomic region. The function *bumphunte*() implements the Bioconductor package *bumphunter* [33] and focuses on short-range (1–2 kb) DNAm changes, e.g. around gene promoters. To find blocks of differential methylation [34], the function *cpgCollapse*() covers long-range changes in DNAm status as represented by the 170,000 open sea probes on the 450k array.

For estimation of cell-type composition of whole blood samples and correction for this difference in composition the *estimateCellCounts*() function implements an algorithm that utilises a reference dataset of different blood cell types to estimate cell composition in any dataset of whole blood [32].

The most recent update to *minfi* is the functional normalisation algorithm, *preprocessFunnorm*() [22]. This utilises control probes on the bead chip (similar to noob in *methylumi*) to remove technical variation. The control probes act as surrogates representing the unwanted variation, which may include batch effects. In fact, the authors show that replication between experiments are improved even with a batch effect present. The method is expected to be most useful in datasets with large differences between samples (e.g. cancer vs. normal). The authors show that the functional normalisation outperforms within array type II adjustment normalisations by improving the ability to replicate findings between experiments.

### 2.3 *wateRmelon*

The *wateRmelon* Bioconductor package was developed to provide access to the 15 normalisation methods and three performance metrics developed and described by Pidsley et al. [12]. The package provides the functions for loading data in text files or IDAT files and for filtering based on detection *p*-value and bead count per probe.

*wateRmelon* takes advantage of known DNAm patterns that have been associated with genomic imprinting, X-chromosome inactivation (XCI) and also the 65 single nucleotide polymorphisms (SNPs) present on the array to create three independent metrics which can be used to test methods of correction and normalisation. Discrete imprinted differentially methylated regions (iDMRs) are expected to have monoallelic methylation (*β* = 0.5). XCI causes male–female differences on the X-chromosome that lead to females showing at least 50% methylation and males substantially less. Finally, the signal from the SNP probes is expected to cluster into three distinct genotype groups (i.e. AA, AB, BB) and could be used to provide an indication of technical variation between samples. These three natural control sets have been used to create the performance metrics *dmrse_row*(), *genki*() and *seabi*() respectively. Of the 15 normalisation functions implemented in *wateRmelon*, the authors show their function, *dasen*(), to be the best performing.

To enable users to take advantage of the performance metrics and normalisation methods *wateRmelon* works with the R classes needed for the other packages.

### 2.4 *ChAMP*

The Chip Analysis Methylation Pipeline, *ChAMP* [35], is a Bioconductor package that streamlines and automates many of the *minfi* functions for a more inexperienced R user. As such it offers eight functions that can be manipulated by changing parameters if the user wishes to use options other than the default values. One useful feature of the *ChAMP* package is the option to run the entire pipeline with a single function *champ.process*(). The caveats with this function are the time it takes to run and the fact that not all intermediate tables are saved. However, once the user is familiar with the package this offers a quick option for subsequent analyses. The default normalisation in *ChAMP* is BMIQ [9] and there is also the option to run *ComBat* [19] to correct for batch effect related to slide number (Sentrix ID) as long as the study design is not confounded by slide number (an example of this would be all control samples on one slide and test samples on another).

*ChAMP* offers a few modules that are currently unique to *ChAMP.* The DMR hunting algorithm ‘Probe Lasso’ is run using the *champ.lasso*() function. Probe lasso requires differential methylation *p*-values for all probes after filtering (even those that are above the significance threshold) and uses a dynamic window based on genomic features to capture DMRs. This method is described in more detail in this issue (Butcher and Beck). In addition, ChAMP includes a module for copy number alteration analysis using the function *champ.CNA*() [19]. This module utilises the intensity values so users must have either the IDAT files or a table of intensity values.

### 2.5 *RnBeads*

*RnBeads*[36] is another comprehensive package that can analyse sequencing based DNAm data, in addition to 27k and 450k data. Like *ChAMP*, *RnBeads* offers a full pipeline option that runs through all the modules with a single function *rnb.run.analysis*(). Alternatively the workflow can be run in steps with a number of normalisation options (including SWAN, BMIQ, *wateRmelon* methods and *methylumi’s noob* method. A benefit of *RnBeads* is the detailed HTML report that describes the analysis that was done along with results and images including a number of quality control plots.

*RnBeads* includes a module for “Annotation Inference” that includes functions to infer gender of samples using an internal classifier based on signal intensity or identify hidden confounders using SVA. This module also implements both a reference based cell type correction method for blood [32] (also implemented in *minfi*) and a reference free method (also available as a stand-alone package described below) [37]. A separate module allows the user to inspect data for sample traits that might lead to batch effects with a few different plots (principal component analysis, PCA and multidimensional scaling, MDS). *RnBeads* also offers functions for interactions with UCSC to allow ENCODE annotation and data visualisation.

*RnBeads* provides images for inspecting methylation profiles and also conducts differential methylation analysis between sample groups using linear modelling (*limma* package) or regular *t*-tests to compute *p*-values. There is an option for paired analysis. The function for calculating DMRs simply calculates the mean methylation across all probes in the genomic region.

## 3 Additional resources

In addition to the comprehensive packages reviewed here we thought it would be worthwhile to highlight other stand-alone packages that can be incorporated into any analysis pipeline.

### 3.1 Quality control

Although all the packages above include modules for assessing quality control two new packages worth mentioning are *shinyMethyl* [38] and *MethylAid* [39]. Both of these packages are interactive Shiny applications (http://www.rstudio.com/shiny/) that enable users to investigate sample quality through an interactive user interface. The packages each provide slightly different diagnostic plots of the internal control probes present on the array, but both allow users to select thresholds and observe the potential impact of batch effects.

### 3.2 Probe annotation

As described above it is important to filter out probes that have failed during hybridisation. In many studies it may also be beneficial to filter out X- and Y-chromosomes. A few resources are now available that annotate probes hybridising to multiple genomic locations (non-specific probes) or those that overlap with SNPs particularly at the probed CpG dinucleotide [40], [41], [42]. Additionally, the Bioconductor package *Illumina450ProbeVariants.db* [43] is a curated collection of data from the 1000 genomes project [44] that includes minor allele frequency of SNPs derived from four populations (American, African, European or Asian) with details on the SNPs distance from the CpG dinucleotide.

### 3.3 DMR calling methods

In addition to the DMR hunting methods included in packages reviewed here (i.e. probe lasso and bumphunter) two stand-alone packages for calling DMRs have been developed recently.

*DMRcate*[45] is a Bioconductor package that uses a kernel function to aggregate groups of significant probes where the distance to the next consecutive probe is less than the designated number of nucleotides.

*FastDMA*[46] is a package written in C++/Python that includes a function to preform basic quantile normalisation before analysing samples for DMPs or DMRs. The DMP analysis uses ANCOVA (a generalised linear model combining ANOVA and regression) to compare two linear regression models, one assuming an overall mean across all study groups while the other assumes different means. A *p*-value is calculated and then the false discovery rate is computed to identify significantly differentially methylated probes. The DMR caller then identifies DMRs containing several probes with uniform methylation in predefined regions (promoter or CpG Island) or in arbitrary regions based on a sliding window that looks for more than two significant probes.

Another package *comb-p* [47] has been extended to work with 450k data. It combines previously calculated *p*-values that are spatially correlated resulting in a list of regions with an aggregated and corrected *p*-value assigned to each region.

### 3.4 Data integration

With the large number of EWAS being conducted and the vast amount of publically available data it is important to come up with methods and tools for data integration. This is an area of analysis that needs more emphasis as data integration can be challenging.

*Marmal-aid*[48] is an R-package and database that is frequently updated and currently includes more than 11,000 450k array samples from nearly 200 different tissues and approximately 100 different diseases. This is a powerful tool for integrating smaller datasets to ask different biological questions, to validate results or to observe trends across different samples.

*COHCAP*[49] is a software package that takes methylation data (450k or BS-Seq) that has been preprocessed and identifies CpG islands that show a consistent methylation pattern among CpG sites. This package also integrates with gene expression data to identify CpG islands that potentially regulate downstream gene expression.

### 3.5 Visualisation

All the pipelines and packages discussed here produce a number of images. However, custom high quality images and plots can be created using R/Bioconductor packages including *ggplot2* [50] and *Gviz* [51]. Analysis output from any of the packages mentioned here could also be imported into UCSC Genome Browser or IGV (Integrative Genomics Viewer) for data visualisation.

## 4 Emerging 450K analyses

As reviewed here, there are a number of comprehensive pipelines for 450k array analysis for those with varying skills in R programming and statistics as well as several packages for downstream data analysis. In addition, a number of new packages have recently been released. Here we briefly highlight some of the most recent developments.

### 4.1 Cell composition correction

An important issue in methylation studies is cellular heterogeneity [23]. Whole blood, like many other tissues, is made up of a number of different cell types each with different methylation profiles that can vary in proportion with age or disease status. Houseman et al. [32] developed a statistical method using reference data to accurately estimate relative proportions of differing cell types in whole blood. This method has been incorporated into methods in *minfi* and *RnBeads*. Guintivano et al. [52] have implemented a similar method for particular brain cell types. A reference dataset for each cell type would be the ideal solution and the International Human Epigenome Consortium (IHEC) is attempting to generate this data. Unfortunately it is costly, time consuming and in some cases unrealistic to come up with reference sets for each cell type. As such two methods have recently been developed to deal with this issue without the need for a reference dataset*. EWasher* [53] is a package written in Perl and *RefFreeEWAS* [37] is an R-package. Both can be used alongside any of the other analysis packages. It is important to consider the top down systems view of the study being analysed before applying a cell-composition correction as it may correct away the methylation differences that are of interest. Cell-composition correction may be of different importance in studies with large methylation differences (cancer vs. normal) compared to those with small methylation differences (common disease).

### 4.2 Estimating sample age

A significant amount of work is going into finding DNAm profiles for particular disease states. However one condition every sample has in common is age. Horvath [54] took advantage of this fact by using 8000 samples from 82 27k or 450k datasets to define a DNAm profile consisting of 353 CpG sites that can act as a predictor of age. It is not clear whether these clock CpGs are simply a marker for age or relate to an effector of aging. However, they do highlight interesting questions that may give us more information on tissue aging. For one, the clock systematically overestimates the age of female breast tissue, and conversely heart tissue age seems to be underestimated while the prediction of brain tissue age is extremely accurate. In addition, work with monozygotic and dizygotic twins showed that age acceleration is extremely heritable. The epigenetic clock script is written in R and utilises the *WGCNA* R-package to measure pure age effects. Details about using the epigenetic clock can be found in the supplementary files of the paper.

### 4.3 Hydroxymethylation analysis

The recent development of chemical [55] and enzymatic [56] assays for oxidation of 5-methylcytosine (5mC) enables the discrimination between 5mC and 5-hydroxymethylcytosine (5hmC) and their quantitative measurements in a single workflow. In addition to sequencing-based applications, this has created a new use for the 450k array which has already been successfully translated for the chemical assay into a commercially available kit (TrueMethyl) by CEGX [57]. First attempts indicate that both assays work on the 450k platform (Field et al., submitted, [58]) but as yet no methods specific to 5hmC analysis have been developed. However, judging by the speed this community has addressed such needs in the past, it will not be long before appropriate methods become available and integrated into the pipelines discussed in this review.

## 5 Limitations and conclusions

Despite its popularity and obvious success, the 450k platform has some limitations which cannot be addressed by computational approaches. The coverage of total CpG sites, for instance, is low (only around 2%) which means that some features such as enhancers are only barely or not at all covered. The design of the Infinium assay does not allow the detection of allele-specific DNAm that is important in the context of imprinting and other parent-of-origin effects. Furthermore, the platform is human-specific and thus not suitable for comparative analysis with model organisms such as mice. The mouse reference genome matches only 13,715 probes on the 450k array [59].

Looking forward, however, the 450k platform is likely to remain popular for some time to come and it will be the platform of choice for EWAS for the foreseeable future. This is largely due to the innovative and freely available computational solutions that the community has been developing to streamline the analysis and address the many technical and biological confounders discussed above. Judged on publications indexed in PubMed and data submissions to GEO (www.ncbi.nlm.nih.gov/geo/), the public repository for this type of data, 450k use and analysis was exponentially growing at the time of writing this review.
